# From Sea to Sight: Fucoidan Protects Against Oxidative Damage in Porcine Retina Organ Culture

**DOI:** 10.3390/md24030088

**Published:** 2026-02-24

**Authors:** Leonie Deppe, Philipp Dörschmann, H. Burkhard Dick, Alexa Klettner, Stephanie C. Joachim

**Affiliations:** 1 Experimental Eye Research Institute, University Eye Hospital, Ruhr-University Bochum, In der Schornau 23-25, 44892 Bochum, Germany; leonie.deppe@rub.de (L.D.); burkhard.dick@knappschaft-kliniken.de (H.B.D.); 2 Department of Ophthalmology, University Medical Center, University of Kiel, Arnold-Heller-Str. 3, Haus 25, 24105 Kiel, Germany; philipp.doerschmann@uksh.de (P.D.); alexa.klettner@uksh.de (A.K.)

**Keywords:** glaucoma, fucoidan, oxidative stress, neuroprotection, porcine organ culture model

## Abstract

Degeneration of retinal ganglion cells (RGCs) is a hallmark of glaucoma. As RGCs are vulnerable to oxidative imbalance, anti-oxidative strategies are of significant interest as novel therapeutic targets. Fucoidans, bioactive compounds derived from algae, are known to be anti-oxidative. Hence, we investigated if fucoidans have protective effects in a retina organ culture model. Porcine explants were pre-treated with fucoidan (*Fucus vesiculosus*; FVs) for 0.5 h (10 or 50 µg/mL). Afterwards, damage was induced through H_2_O_2_ (500 µM; 3 h). Four groups were investigated: control, H_2_O_2_, 10 FVs + H_2_O_2_, and 50 FVs + H_2_O_2_. RGCs, glial cells, hypoxic/oxidative, apoptotic, and ferroptotic markers were examined by immunohistology, RT-qPCR, and a caspase assay. H_2_O_2_ led to lower RGC numbers and RBPMS expression levels while FVs prevented this degeneration. An upregulation of glial expressions and more microglia/macrophages were observed in H_2_O_2_ samples, mitigated by FVs. Anti-oxidative genes increased during stress but normalized with FVs. Apoptotic signaling increased while *GPX4* mRNA expression decreased with H_2_O_2_, both restored by FVs. Consequently, RGC loss was prevented through the attenuation of glial activation, inhibition of hypoxic/oxidative stress, and anti-ferroptotic/apoptotic action mediated by FVs. Advancing glaucoma research, this study emphasizes the therapeutic potential of FVs and offers new directions for future research.

## 1. Introduction

Glaucoma is one of the leading causes of blindness worldwide [1]. The global prevalence between the ages of 40 and 80 is estimated at 3.5% and it is predicted that 111.8 million people will be affected by 2040 [2]. This chronic neurodegenerative disease is described by the loss of retinal ganglion cells (RGCs) and the degeneration of the optic nerve [3,4]. In addition to elevated intraocular pressure (IOP), one of the main risk factors for this disease, glaucoma progression involves a range of other critical pathological changes. Myopia, corneal thinning, and low diastolic blood pressure have been identified as potential risk factors for glaucoma. Furthermore, emerging research suggests that modifiable lifestyle factors, including smoking, alcohol consumption, dietary patterns, and physical activity, may contribute to the pathogenesis of the disease [1]. Glaucoma can also occur in individuals with normal IOP. In addition, IOP-lowering treatments cannot always prevent the progression [1,5,6]. To date, there is no cure for glaucoma, and current IOP-lowering treatments only slow down the course of the disease. Hence, it is crucial to continue research to investigate alternative, novel treatment approaches for glaucoma patients.

One goal of ongoing glaucoma research is to find new therapeutical options which can act neuroprotective and thus prevent RGC loss. Therefore, other pathological aspects like oxidative stress, ischemia, hypoxia, excitotoxicity, and immunological processes are assessed in various model systems [7,8]. Rodent models are most commonly employed in such studies [9,10]. However, they only partially reflect the physiological situation in humans. Consequently, the use of other species could be advantageous. In addition, increasing social pressure is calling for the reduction or even the elimination of these conventional animal models. For this reason, the development and implementation of suitable alternatives are urgently needed. One innovative way to avoid the use of laboratory animals is the utilization of porcine eyes as a byproduct of the food industry [11,12,13,14]. Pig eyes offer several advantages for ophthalmic research. In addition to their size and similar morphology, their cellular structures closely resemble those of human eyes [15,16]. Beside many other advantages, these porcine retinal explants can be cultivated and are useful in the field of glaucoma research [7,17,18,19,20,21].

This porcine organ culture model has already been successfully employed by several research groups [8,17] and was also established in our laboratory a few years ago. In previous projects we used hydrogen peroxide (H_2_O_2_) to induce oxidative stress in porcine retina organ cultures. This resulted in RGC loss and promoted microglia and macrophage accumulation, reflecting glaucoma-like neurodegenerative damage [7,17]. In subsequent studies, we investigated extremolytes, an iNOS-inhibitor, coenzyme Q10, and hypothermia as protective interventions in this organ culture damage model [7,20,22,23].

In the current study, we investigated the possible neuroprotective efficacy of fucoidan. This bioactive nutraceutical, extracted from brown algae, is known for its anti-oxidative as well as anti-inflammatory properties [24,25,26]. The most extensively studied species include *Fucus vesiculosus*, *Fucus distichus*, and *Ascophyllum nodosum*, which are rich sources of fucoidan [27,28]. The fucoidan used in this study is an extract derived from *Fucus vesiculosus* (FVs). Fucoidan consists of L-fucose and other sugars such as xylose, mannose, and galactose [26]. Interestingly, recent studies identified an anti-viral efficacy against SARS-CoV-2 [29,30]. In a Parkinson’s disease study, fucoidan demonstrated promising effects on the dopaminergic system in rats. The authors proposed that this neuroprotective function could be linked to an enhanced mitochondrial preservation via the PGC-1α/NRF2 axis [31]. Additionally, this complex polysaccharide has shown protective effects in age-related macular degeneration (AMD) research [32]. Here they used a cell culture model with primary porcine retinal pigment epithelium cells, damaged with lipopolysaccharide, H_2_O_2_, or CoCl_2_. Fucoidan reduced stress-induced gene activation due to inflammation, oxidative stress, and pathological angiogenesis [33]. Also, a fucoidan dependent glutathione peroxidase 4 (GPX4) modulation, a key regulator of ferroptosis, was identified [34].

Now, we investigated whether FVs displays neuroprotective effects against oxidative stress in the porcine retina organ culture. We hypothesize that FVs confer protection and attenuates H_2_O_2_-induced damage. Our findings indicate pronounced neuroprotective effects of FVs on RGCs, which can primarily be attributed to its anti-oxidative and anti-inflammatory properties. This study therefore represents a first attempt to use FVs in a porcine oxidative stress model and provides evidence for its protective potential. As oxidative stress is a key contributor to glaucoma pathogenesis, this study establishes a basis for initial steps towards new therapeutic strategies. The applied model closely reflects the human *in vivo* situation, thereby underscoring the potential relevance of FVs in ophthalmological research.

## 2. Results

### 2.1. FVs Could Counteract the RGC Loss After Oxidative Stress

**Figure 1:**
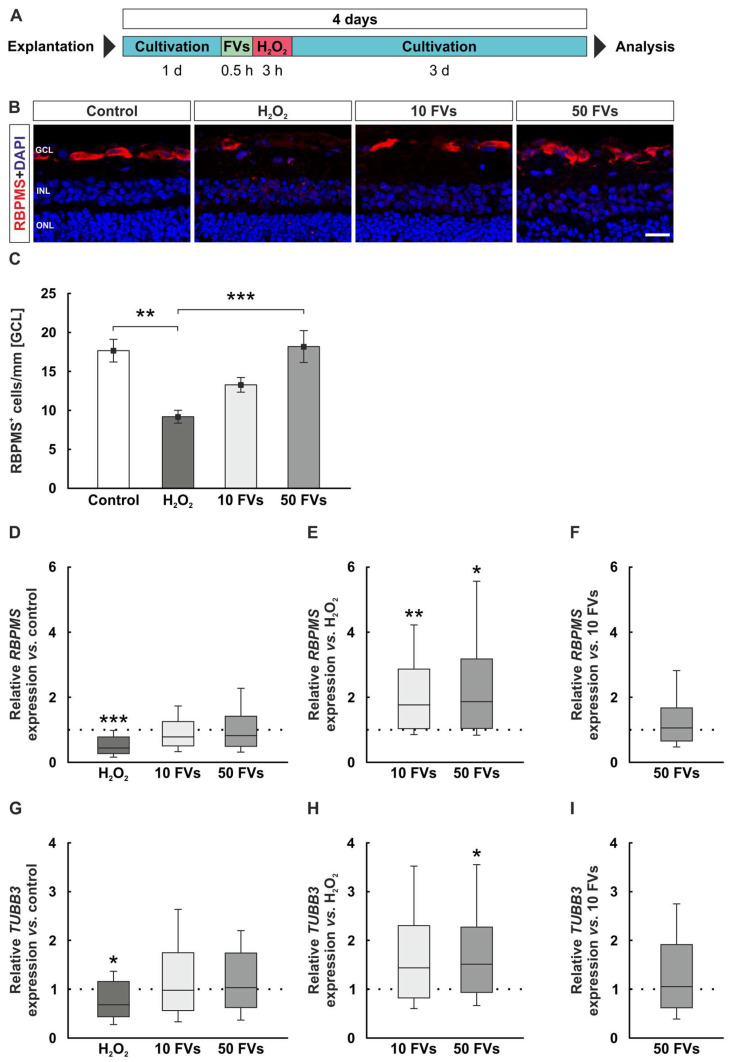
FVs pre-treatment had a protective effect on RGCs exposed to oxidative stress. (**A**) After explantation, retinal tissue was cultivated for one day. FVs pre-treatment (0.5 h) was performed directly before damage induction through H_2_O_2_ (500 µM; 3 h). After three more days, retinal explants were analyzed. (**B**) Representative pictures of RBPMS (red) and DAPI (blue) staining. (**C**) Lower RGC numbers were noted in H_2_O_2_ samples compared to control retinas (*p* = 0.001). This was counteracted by 50 FVs compared to the H_2_O_2_ group (*p* < 0.001). (**D**) The relative mRNA expression of *RBPMS* was downregulated in the H_2_O_2_ tissue (*p* < 0.001). (**E**) The comparison between the FVs tissue and the H_2_O_2_ group showed a *RBPMS* upregulation (10 FVs: *p* = 0.006; 50 FVs: *p* = 0.010). (**F**) There were no differences between both FVs samples in *RBPMS* expression levels. (**G**) The *TUBB3* mRNA expression was downregulated in the H_2_O_2_ retinas (*p* = 0.046). (**H**) Compared to the H_2_O_2_ group, the 50 FVs one displayed a *TUBB3* upregulation (*p* = 0.027). (**I**) There were no differences between both FVs samples. GCL = ganglion cell layer; FVs = *Fucus vesiculosus*; INL = inner nuclear layer; ONL = outer nuclear layer. (**C**): *n* = 9/group, values are shown as mean ± SEM; (**D**–**I**): *n* = 8/group, values are shown as median ± quartile + min/max. Scale bar: 20 µm. * *p* < 0.050, ** *p* < 0.010, and *** *p* < 0.001.

To investigate how RGCs were affected by oxidative stress and the possible protective effects through FVs, a staining against RBPMS (RNA-binding protein with multiple splicing), a RGC-specific marker, and subsequent cell count was performed (Figure 1B). The control retinas displayed 17.66 ± 1.46 cells/mm, while the number of RGCs in the H_2_O_2_ tissue was significantly decreased (9.19 ± 0.83 cells/mm, *p* = 0.001). No changes could be observed in the 10 FVs group compared to controls (13.28 ± 0.94 cells/mm, *p* = 0.143). Also, 50 FVs revealed no differences to controls (18.19 ± 2.05 cells/mm, *p* = 0.993) and significantly prevented the RGC loss compared to H_2_O_2_ samples (*p* < 0.001; Figure 1C).

A decrease in relative *RBPMS* mRNA expression could be detected in the H_2_O_2_ retinas compared to the control samples (0.44-fold expression, *p* < 0.001), while both FVs groups showed no changes (10 FVs: 0.78-fold expression, *p* = 0.166; 50 FVs: 0.82-fold expression, *p* = 0.322; Figure 1D). By comparing the FVs tissue with the H_2_O_2_ ones, the FVs samples illustrated a significant *RBPMS* upregulation (10 FVs: 1.77-fold expression, *p* = 0.006; 50 FVs: 1.87-fold expression, *p* = 0.010; Figure 1E). In the comparison of the two FVs samples, there were no differences (1.06-fold expression, *p* = 0.770; Figure 1F).

*TUBB3* (tubulin beta 3 class III) expression, a marker gene associated with neuronal cells, was downregulated in the H_2_O_2_ retinas compared to control ones (0.68-fold expression, *p* = 0.046) but not in FVs samples (10 FVs: 0.98-fold expression, *p* = 0.922; 50 FVs: 1.04-fold expression, *p* = 0.848; Figure 1G). FVs groups compared to the H_2_O_2_ samples revealed an upregulation of *TUBB3* mRNA expression in the 50 FVs tissue (10 FVs: 1.44-fold expression, *p* = 0.075; 50 FVs: 1.51-fold expression, *p* = 0.027; Figure 1H). There was no alteration in relative *TUBB3* mRNA expression between the two FVs groups (1.05-fold expression, *p* = 0.787; Figure 1I).

### 2.2. Microglia and Macrophage Activation Due to Oxidative Stress Was Reduced with FVs

**Figure 2:**
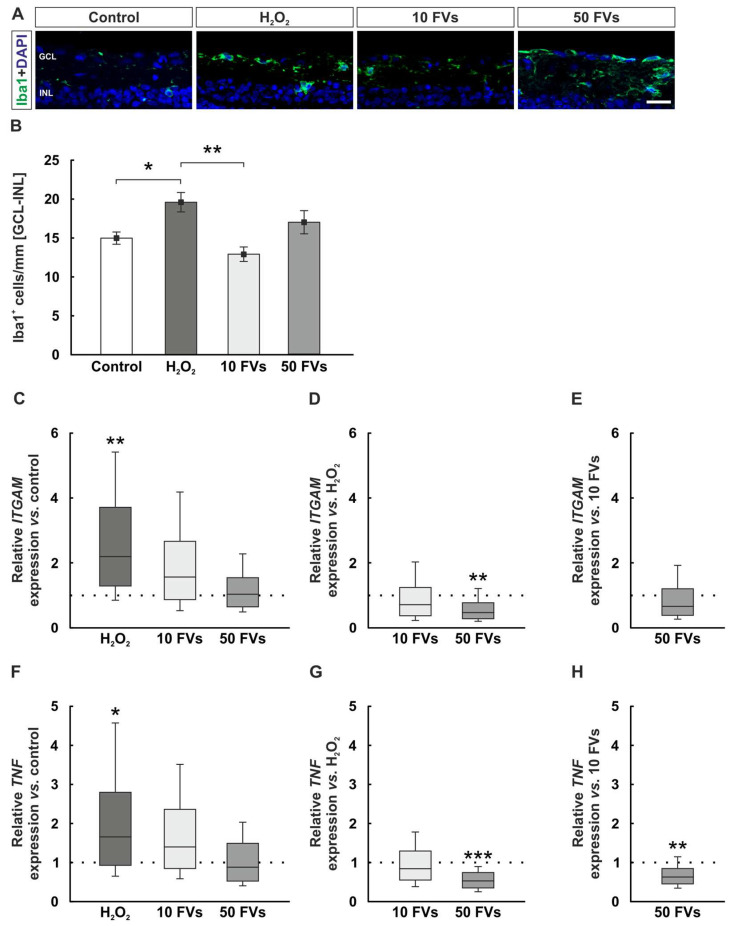
Microglial and macrophage activation following oxidative stress was reduced by FVs. (**A**) Exemplary retinal cross-sections stained with Iba1 (green) and DAPI (blue). (**B**) Cell counts revealed an increased number of Iba1^+^ cells in the H_2_O_2_ group in the GCL-INL (vs. control: *p* = 0.036; vs. 10 FVs: *p* = 0.001). (**C**) The relative *ITGAM* mRNA expression was upregulated in the H_2_O_2_ group compared to control samples (*p* = 0.003). (**D**) A decrease in *ITGAM* expression was observed in the 50 FVs retinas compared to the H_2_O_2_ group (*p* = 0.003). (**E**) No significant changes were observed between the FVs groups. (**F**) *TNF* mRNA levels were upregulated in H_2_O_2_ tissue (*p* = 0.028) compared to controls. (**G**) The 50 FVs group showed a *TNF* downregulation compared to the H_2_O_2_ one (*p* < 0.001). (**H**) The comparison between both FVs groups showed a lower expression of *TNF* in the 50 FVs group (*p* = 0.002). GCL = ganglion cell layer; FVs = *Fucus vesiculosus*; INL = inner nuclear layer. (**B**): *n* = 9/group, values are shown as mean ± SEM; (**C**–**H**): *n* = 8/group, values are shown as median ± quartile + min/max. Scale bar: 20 µm. * *p* < 0.050, ** *p* < 0.010, and *** *p* < 0.001.

Microglia and macrophages were stained with an antibody against Iba1 (ionized calcium-binding adapter molecule 1). Positive cells were observed in the inner retinal layers (Figure 2A). The immunofluoresence analysis showed more Iba1^+^ cells in the ganglion cell layer (GCL) to inner nuclear layer (INL) of H_2_O_2_ retinas (19.60 ± 1.25 cells/mm) compared to the controls (14.99 ± 0.79 cells/mm, *p* = 0.036). No changes were identified in FVs samples in comparison to the controls (10 FVs: 12.92 ± 0.93 cells/mm, *p* = 0.585; 50 FVs: 17.03 ± 1.49 cells/mm, *p* = 0.594). In comparison to the H_2_O_2_ tissue, the 10 FVs group displayed reduced Iba1^+^ cell counts in the GCL-INL (*p* = 0.001; Figure 2B).

The *ITGAM* (integrin alpha M) mRNA expression, which corresponds to CD11b (cluster of differentiation 11b) and is expressed in microglia/macrophages [35,36], was evaluated. *ITGAM* expression was increased in the H_2_O_2_ group compared to controls (2.19-fold expression, *p* = 0.003). Both FVs groups had similar values as the control group (10 FVs: 1.57-fold expression, *p* = 0.054; 50 FVs: 1.04-fold expression, *p* = 0.834; Figure 2C). By comparing the FVs retinas directly with the H_2_O_2_ group, the 50 FVs one displayed a decrease in relative *ITGAM* mRNA expression (10 FVs: 0.72-fold expression, *p* = 0.167; 50 FVs: 0.47-fold expression, *p* = 0.003; Figure 2D). We could not identify significant differences between both FVs groups (0.66-fold expression, *p* = 0.060; Figure 2E).

*TNF* (tumor necrosis factor) gene was investigated to determine the relative mRNA expression levels of cytokine-linked genes. In H_2_O_2_ samples, the expression of *TNF* was significantly upregulated compared to control samples (1.65-fold expression, *p* = 0.028). In the FVs-treated tissue, the expression was comparable to controls (10 FVs: 1.40-fold expression, *p* = 0.099; 50 FVs: 0.88-fold expression, *p* = 0.445; Figure 2F). Compared to H_2_O_2_ retinas, the *TNF* expression in the 50 FVs tissue was downregulated (10 FVs: 0.84-fold expression, *p* = 0.303; 50 FVs: 0.53-fold expression, *p* < 0.001, Figure 2G). The 50 FVs samples displayed lower *TNF* expression levels than the 10 FVs ones (0.63-fold expression, *p* = 0.002; Figure 2H).

### 2.3. Reduced Macroglia Gene Expression with FVs

**Figure 3:**
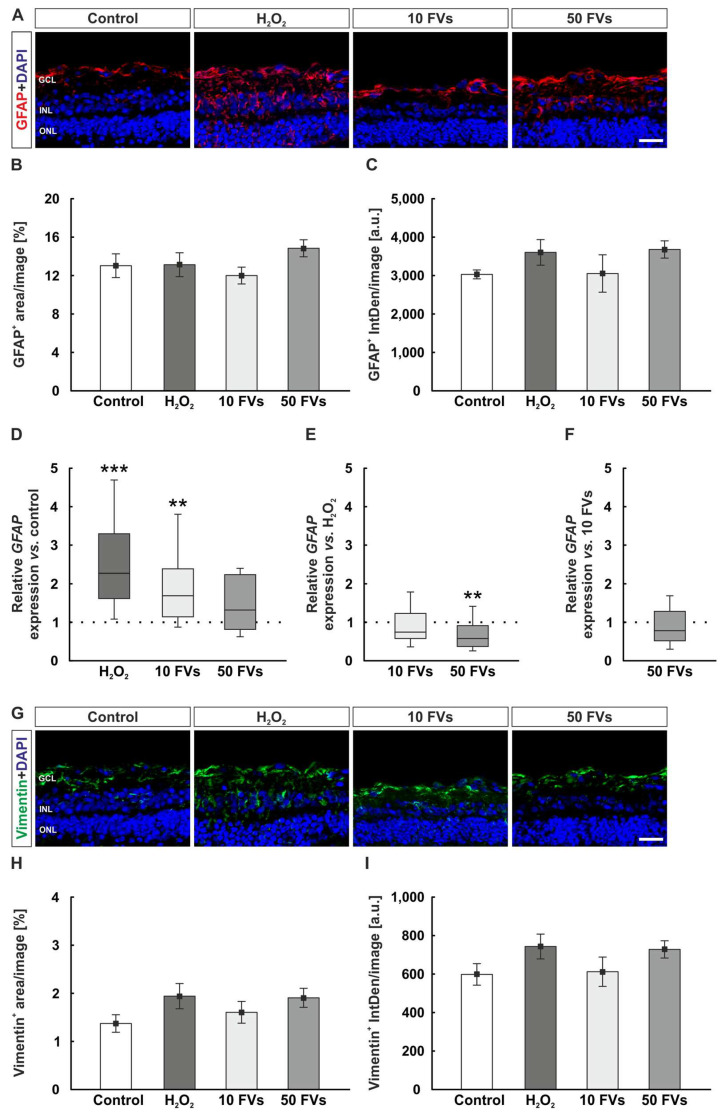
50 FVs samples exhibited a downregulated *GFAP* gene expression. (**A**) Exemplary images of the GFAP staining (red) and DAPI (blue). (**B**,**C**) Area and intensity evaluation of GFAP staining showed no changes. (**D**) An upregulation of relative *GFAP* mRNA expression was observed in the H_2_O_2_ and the 10 FVs samples compared to controls (H_2_O_2_: *p* < 0.001; 10 FVs: *p* = 0.004). (**E**) The 50 FVs group illustrated a downregulation compared to H_2_O_2_ samples (*p* = 0.007). (**F**) The comparison of both FVs groups showed no significant alteration. (**G**) Representative images of the vimentin (green) and DAPI (blue) staining. (**H**,**I**) No changes in the vimentin area and intensity were detected. A.u. = arbitrary units; GCL = ganglion cell layer; FVs = *Fucus vesiculosus*; INL = inner nuclear layer; IntDen = integrated density; ONL = outer nuclear layer. (**B**,**C**,**H**,**I**): *n* = 9/group, values are shown as mean ± SEM; (**D**–**F**): *n* = 8/group, values are shown as median ± quartile + min/max. Scale bars: 20 µm. ** *p* < 0.010 and *** *p* < 0.001.

A staining against GFAP (glial fibrillary acidic protein) and vimentin was performed to investigate macroglia (Figure 3A,G). Regarding the GFAP^+^ area, no significant changes could be observed in all groups compared to control samples (control: 13.03 ± 1.23% area/image; H_2_O_2_: 13.13 ± 1.24% area/image, *p* = 1.000; 10 FVs: 12.00 ± 0.88% area/image, *p* = 0.906; 50 FVs: 14.84 ± 0.89% area/image, *p* = 0.637; Figure 3B). No changes in the GFAP intensity were found within all groups (control: 3030.87 ± 115.97 a.u. IntDen/image; H_2_O_2_: 3603.64 ± 332.95 a.u. IntDen/image, *p* = 0.593; 10 FVs: 3053.59 ± 486.36 a.u. IntDen/image, *p* = 1.000; 50 FVs: 3679.25 ± 224.56 a.u. IntDen/image, *p* = 0.491; Figure 3C).

The *GFAP* mRNA expression in the damaged H_2_O_2_ group was upregulated compared to control retinas (2.27-fold expression, *p* < 0.001). This effect was only reversed in the 50 FVs group, which demonstrated no significant differences to control tissues, while the 10 FV groups showed a significant upregulation (10 FVs: 1.69-fold expression, *p* = 0.004; 50 FVs: 1.32-fold expression, *p* = 0.096; Figure 3D). By comparing both FVs groups with the H_2_O_2_ one, only the 50 FVs samples displayed a downregulation (10 FVs: 0.75-fold expression, *p* = 0.104; 50 FVs: 0.58-fold expression, *p* = 0.007; Figure 3E). A direct comparison of both FVs samples revealed no alteration (0.78-fold expression, *p* = 0.173; Figure 3F).

The vimentin evaluation revealed no changes in vimentin^+^ area within all groups (control: 1.37 ± 0.18% area/image; H_2_O_2_: 1.94 ± 0.26% area/image, *p* = 0.277; 10 FVs: 1.60 ± 0.23% area/image, *p* = 0.876; 50 FVs: 1.91 ± 0.20% area/image, *p* = 0.329; Figure 3H). Also, no variations were detectable regarding the intensity of the vimentin staining (control: 598.34 ± 55.83 a.u. IntDen/image; H_2_O_2_: 743.38 ± 64.47 a.u. IntDen/image, *p* = 0.355; 10 FVs: 612.35 ± 76.17 a.u. IntDen/image, *p* = 0.999; 50 FVs: 728.21 ± 44.71 a.u. IntDen/image, *p* = 0.452; Figure 3I).

### 2.4. Regulation of Hypoxic and Oxidative Stress Genes by FVs

**Figure 4:**
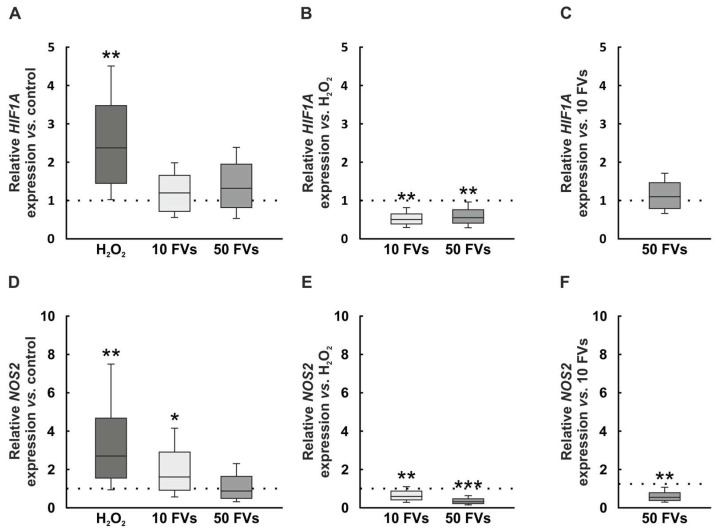
FVs reversed H_2_O_2_-induced upregulation of oxidative and hypoxic stress genes. (**A**) *HIF1A* mRNA expression was upregulated in H_2_O_2_ samples (*p* = 0.001). (**B**) Compared to the H_2_O_2_ group, FVs tissues showed a downregulated *HIF1A* expression (both: *p* = 0.001). (**C**) The FVs retinas showed no group differences. (**D**) *NOS2* mRNA expression was upregulated in the H_2_O_2_ and the 10 FVs group compared to control tissue (H_2_O_2_: *p* = 0.001; 10 FVs: *p* = 0.047). (**E**) *NOS2* expression was reduced in both FVs groups compared to H_2_O_2_ samples (10 FVs: *p* = 0.002; 50 FVs: *p* < 0.001). (**F**) The comparison between both FVs samples displayed a lower *NOS2* expression in the 50 FVs samples (*p* = 0.001). FVs = *Fucus vesiculosus*. *n* = 8/group, values are shown as median ± quartile + min/max. * *p* < 0.050, ** *p* < 0.010, and *** *p* < 0.001.

The hypoxic-associated gene *HIF1A* (hypoxia-inducible factor 1 alpha) mRNA expression was increased in the H_2_O_2_ group compared to control samples (2.37-fold expression, *p* = 0.001). With FVs pre-treatment this effect was no longer visible (10 FVs: 1.20-fold expression, *p* = 0.238; 50 FVs: 1.32-fold expression, *p* = 0.104; Figure 4A). In comparison to the H_2_O_2_ group, both FVs groups displayed a downregulated relative *HIF1A* mRNA expression (10 FVs: 0.51-fold expression, *p* = 0.001; 50 FVs: 0.56-fold expression, *p* = 0.001; Figure 4B). No alteration could be detected by comparing the 50 FVs samples to the 10 FVs group (1.10-fold expression, *p* = 0.372; Figure 4C).

In the H_2_O_2_ group, the relative mRNA expression level of *NOS2* (nitric oxide synthase 2), the equivalent gene in porcine tissue for the inducible nitric oxide synthase (iNOS), was higher than in the control group (H_2_O_2_: 2.70-fold expression, *p* = 0.001). Also, the expression was upregulated in 10 FVs samples, while 50 FVs tissue did not reflect any changes (10 FVs: 1.61-fold expression, *p* = 0.047; 50 FVs: 0.87-fold expression, *p* = 0.525; Figure 4D). *NOS2* expression in the FVs samples was downregulated in comparison to the H_2_O_2_ group (10 FVs: 0.59-fold expression, *p* = 0.002; 50 FVs: 0.32-fold expression, *p* < 0.001; Figure 4E). The 50 FVs group showed a downregulated *NOS2* expression in comparison to the 10 FVs tissues (0.54-fold expression, *p* = 0.001; Figure 4F).

### 2.5. Impact of Oxidative Stress and FVs on Apoptosis

**Figure 5:**
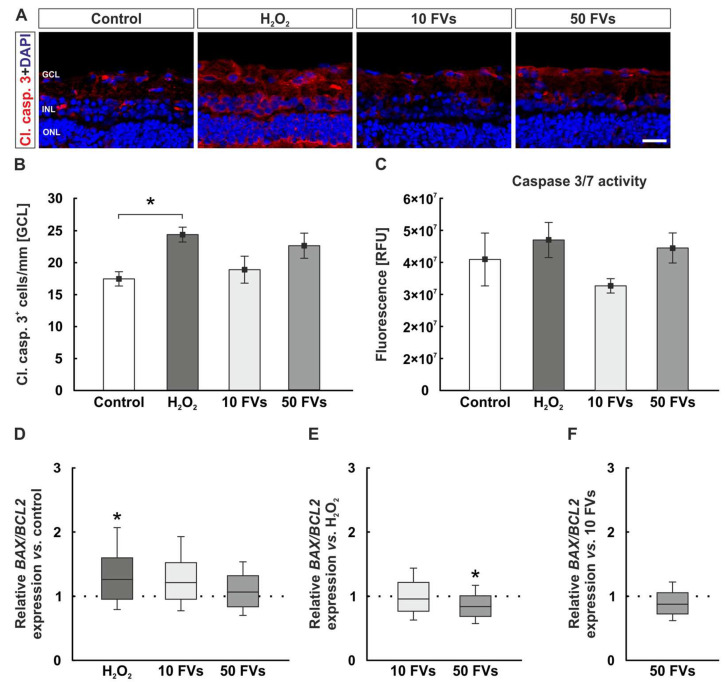
Slight influence of H_2_O_2_ and FVs on apoptosis. (**A**) Representative images showing cl. casp. 3 (red) and DAPI (blue) staining. (**B**) Significantly more cl. casp. 3^+^ cells in the GCL were observed in the H_2_O_2_ samples compared with control retinas (*p* = 0.028). This effect was no longer detectable with FVs treatment. (**C**) The caspase 3/7 assay could not detect any significant changes between all groups. (**D**) With H_2_O_2_ the *BAX*/*BCL2* ratio was upregulated in comparison to the control samples (*p* = 0.029). (**E**) In comparison to the H_2_O_2_ retinas, the 50 FVs samples displayed a downregulated expression (*p* = 0.028). (**F**) The mRNA expression ratio of *BAX*/*BCL2* was not regulated when comparing both FVs groups. GCL = ganglion cell layer; FVs = *Fucus vesiculosus*; INL = inner nuclear layer; ONL = outer nuclear layer; RFU = relative fluorescence units. (**B**): *n* = 9/group, values are shown as mean ± SEM. (**C**): *n* = 7/group, values are shown as mean ± SEM. (**D**–**F**): *n* = 8/group, values are shown as median ± quartile + min/max. * *p* < 0.050.

To evaluate apoptotic signaling, all samples were stained with an antibody against cl. casp. 3 (cleaved caspase 3) to visualize caspase 3 in retinal cells in the GCL. A subsequent cell count of positive cells was performed (Figure 5A). Control retinas exhibited 17.47 ± 1.11 cells/mm. In contrast, cl. casp. 3 counts were significantly higher in H_2_O_2_ tissue (24.40 ± 1.16 cells/mm; *p* = 0.028). No significant differences were detected in the 10 FVs group compared with controls (18.91 ± 2.11 cells/mm; *p* = 0.926). Likewise, treatment with 50 FVs showed no deviation from control values (22.66 ± 1.97 cells/mm; *p* = 0.139; Figure 5B).

Additionally, a caspase 3/7 assay was performed. Here, the control tissue showed 4.09 × 10^7^ ± 0.82 × 10^7^ relative fluorescence units (RFU), while the H_2_O_2_ group displayed 4.70 × 10^7^ ± 0.55 × 10^7^ RFU (*p* = 0.868). There were no statistical changes in the FVs samples, when compared to controls (10 FVs: 3.27 × 10^7^ ± 0.22 × 10^7^ RFU, *p* = 0.727; 50 FVs: 4.45 × 10^7^ ± 0.47 × 10^7^ RFU, *p* = 0.968; Figure 5C).

The ratio of the pro- and anti-apoptotic genes *BAX* (Bcl-2-associated X protein) and *BCL2* (B-cell lymphoma 2) was evaluated. The *BAX*/*BCL2* ratio was upregulated in the H_2_O_2_ group in comparison to control samples (H_2_O_2_: 1.26-fold expression, *p* = 0.029; 10 FVs: 1.21-fold expression, *p* = 0.060; 50 FVs: 1.06-fold expression, *p* = 0.462; Figure 5D). The comparison of the FVs tissue with the damaged one showed a downregulation in the 50 FVs samples (10 FVs: 0.96-fold expression, *p* = 0.617; 50 FVs: 0.84-fold expression, *p* = 0.028; Figure 5E). When comparing the FVs retinas, no significant alteration was noted (0.88-fold expression, *p* = 0.079; Figure 5F).

### 2.6. Modulation of Anti-Oxidative Systems by H_2_O_2_ and FVs

**Figure 6:**
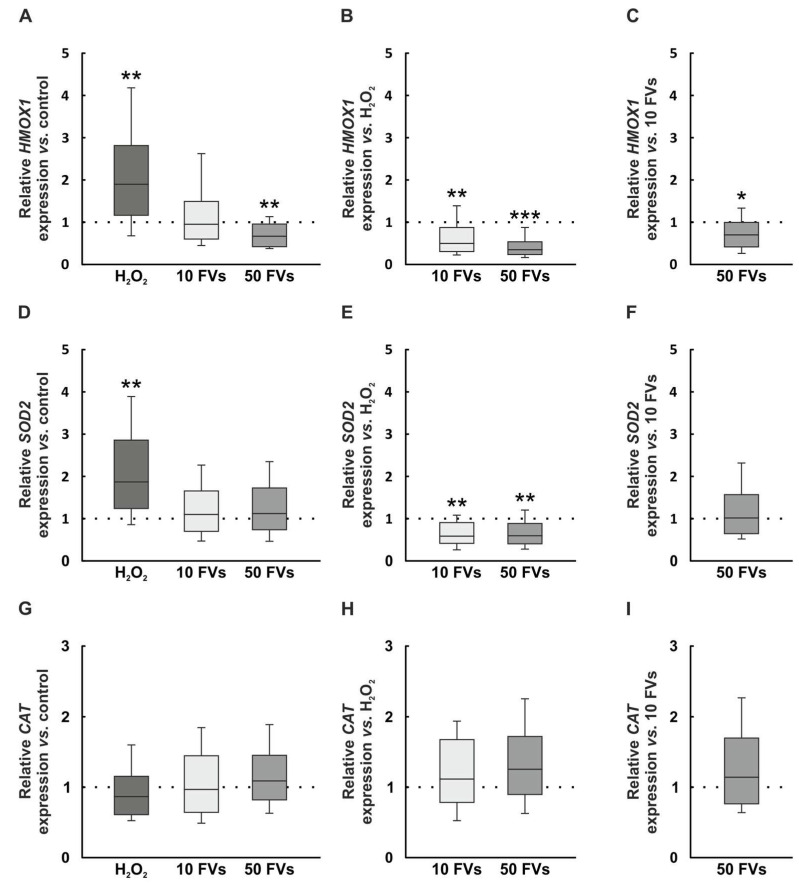
Anti-oxidative systems are differently regulated. (**A**) The relative *HMOX1* mRNA expression was upregulated by H_2_O_2_ (*p* = 0.003) and downregulated with 50 FVs (*p* = 0.009) in comparison to control samples. (**B**) By comparing the FVs groups to the H_2_O_2_ group, both revealed a downregulated *HMOX1* expression (10 FVs: *p* = 0.003; 50 FVs: *p* < 0.001). (**C**) The comparison between both FVs retinas revealed a downregulated expression in the 50 FVs tissues (*p* = 0.026). (**D**) The *SOD2* mRNA levels were upregulated in the H_2_O_2_ tissues compared to control samples (*p* = 0.002). (**E**) The *SOD2* expression of the FVs samples showed a downregulation compared to the H_2_O_2_ group (10 FVs: *p* = 0.004; 50 FVs: 0.007). (**F**) The FVs tissues exhibited comparable results. (**G**) All groups showed similar values regarding the *CAT* expression. (**H**) The FVs groups did not display an alteration when compared with the H_2_O_2_ one. (**I**) No differences were visible between both FVs samples. FVs = *Fucus vesiculosus*. *n* = 8/group, values are shown as median ± quartile + min/max. * *p* < 0.050, ** *p* < 0.010, and *** *p* < 0.001.

In order to characterize the intrinsic anti-oxidative defense, specific genes were examined. The mRNA level of *HMOX1* (heme oxygenase 1), which encodes the protein heme oxygenase 1 (HO-1) and is associated with the nuclear factor erythroid-2-related factor 2 (Nrf2), was upregulated after H_2_O_2_ exposure (1.90-fold expression, *p* = 0.003). The addition of 10 FVs did not reveal an alteration (0.95-fold expression, *p* = 0.761). In contrast, the 50 FVs group showed a downregulation of *HMOX1* compared to control retinas (0.66-fold expression, *p* = 0.009; Figure 6A). FVs tissue displayed a downregulation of *HMOX1* compared to the H_2_O_2_ group (10 FVs: 0.50-fold expression, *p* = 0.003; 50 FVs: 0.35-fold expression, *p* < 0.001; Figure 6B). By comparing the FVs samples, the higher FVs concentration showed a downregulated *HMOX1* expression (0.70-fold expression, *p* = 0.026; Figure 6C).

The relative *SOD2* (superoxide dismutase 2) mRNA expression, part of the mitochondrial anti-oxidative defense system and Nrf2-related, was upregulated in the H_2_O_2_ group (1.87-fold expression, *p* = 0.002). No changes were observable in the FVs samples compared to the control situation (10 FVs: 1.10-fold expression, *p* = 0.566; 50 FVs: 1.12-fold expression, *p* = 0.504; Figure 6D). Relative to the H_2_O_2_ group, the FVs tissues showed a downregulation of *SOD2* mRNA levels (10 FVs: 0.59-fold expression, *p* = 0.004; 50 FVs: 0.60-fold expression, *p* = 0.007; Figure 6E). The 50 FVs retinas did not differ from the 10 FVs ones (1.02-fold expression, *p* = 0.915; Figure 6F).

The relative *CAT* (catalase) mRNA expression, which is part of the general anti-oxidative defense, displayed no changes compared to control samples (H_2_O_2_: 0.87-fold expression, *p* = 0.232; 10 FVs: 0.97-fold expression, *p* = 0.797; 50 FVs: 1.09-fold expression, *p* = 0.499; Figure 6G). In comparison to the H_2_O_2_ tissue, no alterations in the *CAT* expression were observable in FVs tissues (10 FVs: 1.12-fold expression, *p* = 0.439; 50 FVs: 1.26-fold expression, *p* = 0.088; Figure 6H). There were no statistical changes regarding *CAT* when comparing the FVs groups (1.13-fold expression, *p* = 0.402; Figure 6I).

### 2.7. Only the Anti-Ferroptotic GPX4 Gene Expression Varies with H_2_O_2_ and FVs

**Figure 7:**
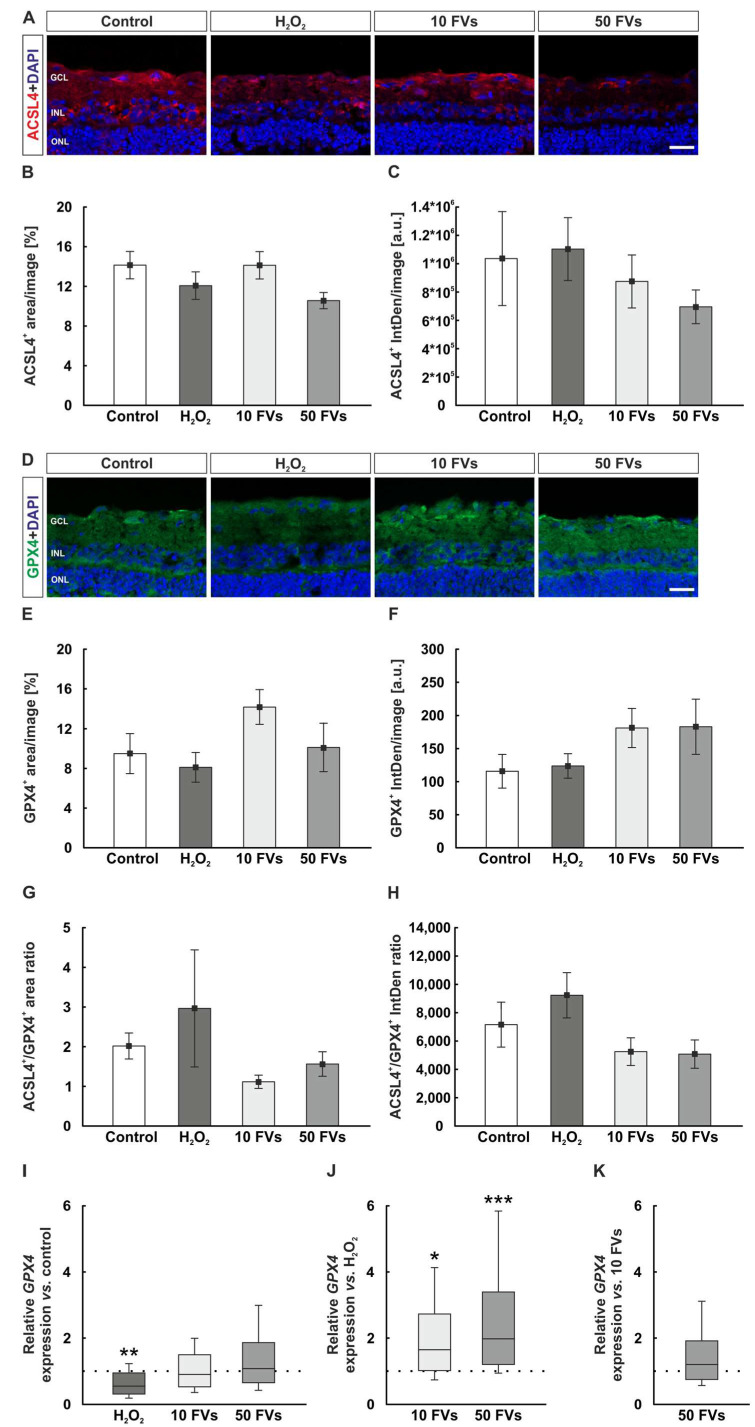
Reduced anti-ferroptotic *GPX4* expression after H_2_O_2_ rescued via FVs. (**A**) Representative images of retinal cross sections stained against ACSL4 (red) and DAPI (blue). (**B**) The ACSL4^+^ area was not altered across all groups. (**C**) No significant changes could be observed by comparing the ACSL4 intensity. (**D**) Exemplary staining of GPX4 (green) and DAPI (blue) on retinal cross-sections. (**E**) The GPX4^+^ area was comparable within the experimental groups. (**F**) The fluorescence intensity of GPX4 was unchanged. (**G**) As a ferroptotic ratio, the positive area of ACSL/GPX4 was analyzed. No significant changes were detectable. (**H**) ACSL4/GPX4 fluorescent intensity ratio did not differ among the groups. (**I**) The relative *GPX4* mRNA expression was downregulated in the H_2_O_2_ tissue compared to the control situation (*p* = 0.008). (**J**) *GPX4* expression levels in the FVs groups were upregulated in comparison to H_2_O_2_ samples (10 FVs: *p* = 0.013; 50 FVs: *p* < 0.001). (**K**) No changes were visible between the FVs groups. A.u. = arbitrary units; GCL = ganglion cell layer; FVs = *Fucus vesiculosus*; INL = inner nuclear layer; IntDen = integrated density; ONL = outer nuclear layer. (**B**,**C**,**E**–**H**): *n* = 9/group, values are shown as mean ± SEM. (**I**–**K**): *n* = 8/group, values are shown as median ± quartile + min/max. Scale bars: 20 µm. * *p* < 0.050, ** *p* < 0.010, and *** *p* < 0.001.

Ferroptotic markers were also investigated. The positive area of the pro-ferroptotic enzyme ACSL4 (Acyl-CoA-synthetase long-chain family member 4) was analyzed on stained retinal cross-sections (Figure 7A). With respect to the ACSL4^+^ area, no significant changes were detected among the groups (control: 14.14 ± 1.38% area/image; H_2_O_2_: 12.07 ± 1.39% area/image, *p* = 0.658; 10 FVs: 14.13 ± 1.38% area/image, *p* = 1.000; 50 FVs: 10.57 ± 0.82% area/image, *p* = 0.210; Figure 7B). Likewise, ACSL4 fluorescence intensity remained unchanged across all conditions (control: 1.04 × 10^6^ ± 3.32 × 10^5^ a.u. IntDen/image; H_2_O_2_: 1.10 × 10^6^ ± 2.22 × 10^5^ a.u. IntDen/image, *p* = 0.997; 10 FVs: 8.75 × 10^5^ ± 1.87 × 10^5^ a.u. IntDen/image, *p* = 0.958; 50 FVs: 6.96 × 10^5^ ± 1.19 × 10^5^ a.u. IntDen/image, *p* = 0.719; Figure 7C).

GPX4, another anti-oxidative marker, which is also known to be anti-ferroptotic, was used to stain porcine retina (Figure 7D). Evaluation of GPX4 revealed no significant differences in the positive area across all experimental groups (control: 9.50 ± 2.02% area/image; H_2_O_2_: 8.11 ± 1.50% area/image, *p* = 0.958; 10 FVs: 14.19 ± 1.74% area/image, *p* = 0.342; 50 FVs: 10.12 ± 2.44% area/image, *p* = 0.996; Figure 7E). Similarly, GPX4 fluorescence intensity did not differ significantly between all conditions (control: 115.79 ± 25.47 a.u. IntDen/image; H_2_O_2_: 123.76 ± 18.51 a.u. IntDen/image, *p* = 0.998; 10 FVs: 181.17 ± 29.55 a.u. IntDen/image, *p* = 0.427; 50 FVs: 183.10 ± 41.77 a.u. IntDen/image, *p* = 0.401; Figure 7F).

Afterwards, the ferroptotic ratio of ACSL4/GPX4 was calculated. The area ratio revealed no alterations among all experimental groups (control: 2.02 ± 0.33 area ratio; H_2_O_2_: 2.97 ± 1.48 area ratio, *p* = 0.823; 10 FVs: 1.12 ± 0.17 area ratio, *p* = 0.844; 50 FVs: 1.56 ± 0.31 area ratio, *p* = 0.976; Figure 7G). Analogously, the ACSL4/GPX4 fluorescence intensity ratio did not differ between any of the conditions (control: 7158.20 ± 1587.58 IntDen ratio; H_2_O_2_: 9229.81 ± 1597.55 IntDen ratio, *p* = 0.689; 10 FVs: 5251.63 ± 975.17 IntDen ratio, *p* = 0.740; 50 FVs: 5076.77 ± 997.40 IntDen ratio, *p* = 0.686; Figure 7H).

RT-qPCR analysis revealed a downregulation of *GPX4* mRNA expression in H_2_O_2_ samples (0.54-fold expression, *p* = 0.008). With FVs pre-treatment this effect was no longer visible (10 FVs: 0.89-fold expression, *p* = 0.559; 50 FVs: 1.07-fold expression, *p* = 0.724; Figure 7I). The comparison of the FVs groups with the H_2_O_2_ group illustrated an upregulation of relative *GPX4* mRNA expression (10 FVs: 1.65-fold expression, *p* = 0.013; 50 FVs: 1.98-fold expression, *p* < 0.001; Figure 7J). There were no alterations between FVs samples (1.20-fold expression, *p* = 0.326; Figure 7K).

## 3. Discussion

Treatment strategies for glaucoma have largely focused on reducing the IOP, while the development of effective neuroprotection remains unsolved to date [37,38]. In this study we used a porcine *ex vivo* organ culture system, mimicking glaucoma-like damage through the induction of neurodegeneration by H_2_O_2_ [7,17,20]. The resulting oxidative stress should be counteracted by an anti-oxidative agent. Therefore, we used FVs pre-treatment combined with oxidative stress to investigate its possible anti-oxidative and protective properties. Our study demonstrated a protective effect of FVs on RGCs through microglia and macrophage inhibition. Moreover, it revealed a distinct oxidative expression profile and indicated a potential regulatory influence via apoptosis and ferroptosis modulation.

Fucoidans are complex and heterogenous polysaccharides extracted from brown algae. Their structural complexity can vary depending on the species, extraction method, and molecular weight. They are primarily composed of L-fucose and sulfate groups but may also contain mannose, galactose, glucose, xylose, uronic acids, acetyl groups, and proteins [39]. Due to their polarity, fucoidans are water-soluble, and the negatively charged sulfate groups allow them to form ionic complexes with oppositely charged molecules. This property facilitates the formation of structured networks in combination with other polymers [40,41]. The *Fucus vesiculosus*-derived fucoidan used in this work has been chemically characterized and reported in earlier publications [34]. With a molecular weight of 52 kDa, it is classified as a high-molecular-weight fucoidan. Such fucoidans are known to exhibit anti-inflammatory [42,43], anti-angiogenic [44], and anti-oxidative [45] properties. Although fucoidans are able to scavenge free radicals extracellularly through the sulfate and hydroxyl groups, this mode of action is more commonly found in low-molecular weight fucoidans, as the binding sites are more exposed [46]. Thus, the observed effects are more likely attributable to receptor interactions [47] or clathrin-mediated endocytosis [48]. These mechanisms of action may trigger a signaling cascade that affects Nrf2, GPX4, or mitochondria-associated processes [34].

In the current study, we observed RGC loss after oxidative stress induction through H_2_O_2_, while pre-treatment with FVs could prevent this damage. These findings are consistent with the assumption that oxidative stress triggers a reactive oxygen species (ROS) overproduction, leading to the degeneration of neuronal cells [49]. RGCs are highly sensitive to a disrupted homeostasis and the presence of ROS [50,51]. The number of RGCs was not significantly decreased with FVs, suggesting sufficient protection. One possible explanation is the enhancement of mitochondrial function. Mitochondria are closely linked to oxidative stress [52]. ROS damages mtDNA, mitochondrial lipid membranes, and interrupts the respiratory chain complex, leading to dysfunction and apoptosis [53]. A positive effect via fucoidan on mitochondria has already been demonstrated in dopaminergic neurons within a rat model regarding Parkinson’s disease. Here, they used a fucoidan from *Laminaria japonica* after rotenone-induced degeneration. It reversed the loss of dopaminergic neurons due to the preservation of mitochondrial respiratory function [31]. It can therefore be assumed that pre-treatment with FVs reduced oxidative stress levels and stabilized mitochondria, resulting in RGC protection.

The anti-inflammatory function of FVs also appears to have played a major role in cell protection, since inflammation can influence cell death [24,26]. H_2_O_2_ led to an increase in microglia and macrophage occurrence. However, this effect was no longer apparent in samples that underwent FVs pre-treatment. These findings further support the anti-inflammatory properties of FVs in retinal tissue, consistent with earlier reports [54]. In line with these observations, FVs appeared to reduce the activity of toll-like receptor 4-activated microglia in primary porcine cell cultures, as reflected by cell size and alterations in microglial phenotype [55]. In general, fucoidan inhibits the pro-inflammatory NF-κB and MAPK signaling pathway, thereby attenuating microglia activation [56]. Supporting the study mentioned above, evidence suggests that fucoidan can block toll-like receptor signaling, leading to a decreased production of pro-inflammatory mediators such as TNFα [57,58]. Subsequently, *TNF* expression was examined in our current study, since oxidative stress can activate immunological reactions [17]. TNFα itself is a pro-inflammatory cytokine and can induce apoptotic cell death via caspases [59]. It is secreted by microglia and regulates the neuroinflammatory injury response [60]. In line with this, an upregulation in *TNF* mRNA expression could be observed. The observed downregulation in the FVs pre-treated groups compared to the damaged group supports the idea of an anti-inflammatory effect due to FVs, possibly corresponding to changing microglia/macrophage occurrence. A similar regulatory effect was observed in a macrophage cell culture model, where fucoidan appeared to downregulate LPS-induced expression of pro-inflammatory cytokines [61]. As both overactivation and insufficient microglia activity can harm the tissue, predicting exact consequences is complex [62]. However, the literature mostly aligns with our results and supports the hypothesis that the observed RGC protection may have been mediated through an anti-inflammatory mechanism.

The histological analysis of macroglia did not support the observed increase in relative *GFAP* mRNA expression after H_2_O_2_ incubation. Even the FVs pre-treatment itself did not cause any changes. GFAP, a key protein in macroglia, is typically an indicator of gliosis and can occur during progressive neurodegeneration [63,64,65]. Other studies using porcine *ex vivo* models demonstrated a modest macroglia reaction due to oxidative stress [17]. It appears that both the type of injury and the evaluation time point are crucial for macroglia reaction. Relatively little is known about the influence of FVs on macroglia. However, as this model does not exhibit excessive macrogliosis, it is unlikely that FVs exerts its protective function through this signaling pathway.

The transcriptional response to hypoxia is mediated by HIFs, which affect glaucoma pathology [66,67]. In donor eyes of glaucoma patients, higher expression of HIF-1 in RGCs were detected [68]. Similar results were discovered in a porcine organ culture model, where more HIF1α^+^ cells were accompanied by a significant RGC loss after damage induction with CoCl_2_ [22,23]. Accordingly, the upregulation of *HIF1A* in our study appears to be a consequence of H_2_O_2_ damage. Furthermore, other studies underlined the upregulation of iNOS after oxidative stress [17,20]. Hypoxia and oxidative stress are strongly interlinked and interdependent in neurodegenerative diseases [69,70]. Several cell culture studies could demonstrate the downregulating influence of fucoidans on HIF1α and iNOS under stress conditions via inhibiting NF-κB, AP-1, p38, ERK, and PI3K/Akt/mTOR [71,72,73,74]. In conclusion, FVs pre-treatment appears to have effective protection due to its anti-oxidative and anti-hypoxic modulating ability.

Apoptosis is a programmed cell death pathway, and its dysregulation can lead to the development of neurodegenerative disorders [75]. Studies reported increased apoptosis in porcine retinas, e.g., after blue light exposure [76]. Anti-apoptotic effects of fucoidans were seen in neuronal cell culture models, mediated by the reduction of ROS, the inhibition of caspases 9 and 3 [77], by modulation of the Bax/Bcl2 ratio, and activation of the PI3K/Akt pathway via NGF [78]. In the present study, changes could be found in the *BAX*/*BCL2* ratio on mRNA level and in the number of cl. casp. 3^+^ cells, indicating apoptotic signaling after H_2_O_2_ exposure, while no significant alteration was visible in the caspase 3/7 assay. This data indicates that H_2_O_2_-induced stress leads to a shift toward pro-apoptotic signaling, suggesting priming of the intrinsic mitochondrial apoptotic pathway [19,21]. The absence of these alterations in response to FVs pre-treatment aligns with previous reports, supporting the anti-apoptotic role of FVs. Bax and Bcl2 function as upstream regulatory factors that determine the apoptotic fate of the cell and act prior to caspases. In contrast, caspases represent the executioner proteins of apoptosis [79]. At first glance, these findings may appear contradictory, as no caspase activity was detected despite changes in the number of cl. casp. 3^+^ cells and the *BAX*/*BCL2* mRNA ratio. However, proper interpretation requires consideration of temporal sequence of apoptotic events, the kinetics following cellular damage, and the specific characteristics of the analytical methods employed. In comparison, mRNA signals are slower to change and considerably more stable over time. A caspase assay measures enzymatic activity in the total retina, which is known to be rapid and transient. Once activated, caspases quickly include cellular fragmentation, after which cells may no longer be detectable or enzymatically active [75]. Furthermore, the retina is a highly heterogeneous tissue, and individual cell types may respond differently to stress. While the immunohistological analysis specifically targeted cells within the GCL, the caspase 3/7 activity assay was performed on whole retina lysates. This methodological difference may have diluted cell-specific effects, thereby reducing the sensitivity of the assay. Overall, our data indicate a pro-apoptotic environment with selective caspase 3 activation, but no strong or synchronous caspase 3/7 activity.

Nrf2, a well-studied transcription factor, regulates the expression of HO-1, SODs, and GPX4 and is activated via oxidative stress [80]. Given this connection, the observed upregulation of *HMOX1* as a Nrf2-associated gene during H_2_O_2_ exposure in this study appears consistent. The subsequent normalization of its expression with FVs indicates an anti-oxidative effect, as oxidative stress no longer acts as a trigger. On the other hand, there is substantial evidence that fucoidans exert an anti-oxidative effect precisely through the activation of Nrf2 and its downstream proteins [81,82]. This would contradict the observed effects, as higher concentrations of FVs even led to a downregulation of *HMOX1*. This is why an analysis of Nrf2 on protein level would be beneficial. SODs are anti-oxidative enzymes and can protect against ROS [83,84]. SOD2 works mainly in the mitochondrial matrix and is strongly correlated to the energy supply during oxidative stress [85,86]. In neurodegenerative diseases, oxidative stress impairs mitochondria and leads to a deficiency of ATP [87]. Since *SOD2* expression is activated by various inflammatory cytokines such as interleukin (IL)-1, IL-6, TNFα, or interferon-γ, and we also observed elevated *TNF* expression, the increase in *SOD2* expression is therefore consistent and verifiable [88,89]. The FVs pre-treatment inhibited *SOD2* expression, suggesting effective mitochondrial protection. However, murine *in vivo* studies have shown an increase in SODs at protein level with a fucoidan in CA1 pyramidal neurons [90]. It would be beneficial to examine the protein level as well. We also investigated catalase, a H_2_O_2_-metabolizing enzyme, localized in the matrix of peroxisomes and cytosol. The transfer of catalase via BAK enables the cell to control local H_2_O_2_ peaks and oxidative stress [91]. In our study, H_2_O_2_ and FVs did not cause any influence on *CAT* expression.

GPX4, a protective enzyme, reduces complex lipid hydroperoxides into lipid alcohols, using glutathione [92,93], thereby acting as a central regulator of ferroptosis, an iron-dependent form of cell death. Its inhibition triggers the activation of this specific pathway [94,95,96]. In our study, H_2_O_2_ led to a reduction in *GPX4* mRNA expression, but the GPX4 staining could not reflect these results. The altered mRNA expression may be due to a suppression of the Nrf2/ARE pathway, as shown previously in primary cardiomyocytes [97]. Although ferroptosis has not yet been systemically investigated in our model, this evidence renders the observed modulation of *GPX4* mRNA expression particularly noteworthy. Another study investigating a cellular model of placental oxidative stress demonstrated that H_2_O_2_ induces downregulation of GPX4 [98]. In line with these findings, research on AMD showed that fucoidan is able to maintain GPX4 in ARPE-19 cells [34]. Similarly, in a mouse model, fucoidan alleviated doxorubicin-induced cardiotoxicity by inhibiting ferroptosis via the Nrf2/GPX4 signaling pathway [99]. The precise mechanism by which FVs reverses the inhibition of GPX4 in our model remains to be fully elucidated. To obtain a more comprehensive view of the potential involvement of ferroptosis in our model, we additionally examined the pro-ferroptotic marker ACSL4. ACSL4 catalyzes the esterification of polyunsaturated fatty acids, thereby promoting the incorporation of them into membrane phospholipids and increasing cellular susceptibility to ferroptosis [100]. However, immunohistological staining for ACSL4 did not reveal any significant differences between experimental groups. Also, the ACSL4/GPX4 ratio did not show a statistically significant difference between groups. However, Nrf2 and ferroptosis represent promising targets for further research, particularly with regard to potentially preventing cell death via the use of ferroptosis-inhibitors [101].

While the discussed findings provide valuable insights, it is important to acknowledge certain limitations of this study that should be considered when interpreting the results. First, an FVs-only control group for comparison was not included. This approach was based on preliminary data indicating no toxic effects of FVs alone. As the study was designed to assess FVs within a retina organ culture model, inclusion of this group was not expected to yield additional insights or contribute substantially to addressing the research question. Nevertheless, the absence of this group should be recognized as a limitation. Second, additional cell death pathways, such as necroptosis or autophagy, were not investigated. As the present study focused on FVs-mediated protection of RGCs, multiple signaling pathways were examined, including apoptosis, inflammation, ferroptosis, as well as oxidative and hypoxic stress. However, other potentially relevant cellular mechanisms may also contribute to the observed effects. The investigation of these aspects was beyond the scope of this study and should therefore be considered in future studies.

## 4. Materials and Methods

### 4.1. Preparation of Explants, Cultivation, and FVs Pre-Treatment

The porcine eyes used were obtained from a local slaughterhouse (registration number: DE05911002921). The tissue was prepared as described previously [7,21]. Briefly, eye cups were opened, cleaned, and cut into quarters. A piece of retina was punched out of each quarter (Ø 6 mm, KAI Medical, KAI Industries Co., Ltd., Gifu, Japan) and placed on a filter insert (Merck Millipore, Burlington, MA, USA). 1 mL of Neurobasal A medium, supplemented with 0.8 mM L-glutamine, 2% B27, 1% N2 (all Gibco^®^ Thermo Fisher Scientific, Waltham, MA, USA), and 2% penicillin/streptomycin (Sigma-Aldrich, St. Louis, MO, USA), was used. The cultivation was performed for four days in 6-well plates (FALCON^®^, Corning Ing., Corning, New York, NY, USA) at 37 °C and in a 5% CO_2_ atmosphere. The chemically characterized, commercially available fucoidan from *Fucus vesiculosus* (FVs, Sigma Aldrich; Cat-No: #F5631; Batch-No: SLBT5471) was used in this study [34]. According to the manufacturer, the brown algae for this batch were harvested from the Canadian coastline. FVs is typically found in cold, nutrient-rich waters, such as the North Atlantic and the eastern and northern Baltic Sea, where it grows in the littoral zone [102]. FVs was added one day after explantation (10 µg/mL, 50 µg/mL) for 0.5 h [54]. Afterwards, 500 µM H_2_O_2_ (Sigma Aldrich) was added to the medium for 3 h. Four experimental groups were studied: control, H_2_O_2_, 10 µg/mL FVs + H_2_O_2_ (10 FVs), and 50 µg/mL FVs + H_2_O_2_ (50 FVs).

After four days of cultivation, the explants for immunohistology (*n* = 9/group) were fixed, embedded and stored at −80 °C. For RT-qPCR (*n* = 8/group), samples were frozen at −80 °C, whereas the samples for the caspase 3/7 assay (*n* = 7/group) were processed immediately (Figure 1A).

### 4.2. Immunohistological Staining and Evaluation

For immunohistological analysis (*n* = 9/group), retinal explants were cut into cross-sections (10 µm). These were stained with primary antibodies (Table 1) against RBPMS, Iba1, GFAP, vimentin, cl. casp. 3, ACSL4, and GPX4 [7,23]. The blocking solution consisted of 10–20% normal donkey serum, 1% bovine serum albumin, and 0.1–0.2% TritonX diluted in 1 × PBS. Primary antibodies were diluted in blocking solution and applied at room temperature overnight. The next day, secondary antibodies (Table 1) were diluted and incubated light protected for 1 h. To visualize cell nuclei, 4’,6’-Diamidin-2-phenylindol (DAPI, SERVA Electrophoresis, Heidelberg, Germany; 1:10) was used. Using a fluorescence microscope (Axio Imager M2, Zeiss, Oberkochen, Germany), four images per cross-section were captured (400× magnification). Images were cropped to a resolution of 800 × 600 pixel (Corel PaintShop Pro X8, Corel, Ottawa, ON, Canada).

Regarding RBPMS, Iba1, and cl. casp. 3, positive signals were counted. RBPMS and cl. casp. 3 positive cells were counted in the GCL and Iba1 positive cells in the GCL-INL. GFAP, vimentin, ACSL4, and GPX4 staining were evaluated with an area analysis. The software ImageJ (ImageJ 1.44 M; NIH, Bethesda, MD, USA) was used for both. Images were analyzed in a blinded manner. Signals co-localized with DAPI were counted. For the area evaluation, the signal area/intensity from GCL to the outer nuclear layer (ONL) was identified using an ImageJ macro. Beforehand, the images were converted into 32-bit grey scale and the background was subtracted (rolling ball (all): 50 pixels; GFAP: lower threshold = 15.61; upper threshold = 85.00; vimentin: lower threshold = 19.91; upper threshold = 43.00; ACSL4: lower threshold = 8.27; upper threshold = 84.85; GPX4: lower threshold = 4.83; upper threshold = 42.67) [23,103].

### 4.3. Quantitative Real-Time PCR (RT-qPCR)

The mRNA was isolated and transcribed into cDNA. The mRNA isolation (*n* = 8/group) was performed using the GeneEluteTM Mammalian Total RNA Miniprep Kit (Sigma-Aldrich) according to manufacturer’s instructions. The final mRNA concentration could be measured using the NanoDropTM One spectrophotometer (Thermo Fisher Scientific). cDNA synthesis was carried out using 1 µg of mRNA and the First Strand cDNA Synthesis Kit (Thermo Fisher Scientific). RT-qPCR analysis was performed following the SYBR Green I protocol with a PikoReal^TM^ 96 Real time Thermal Cycler (Thermo Fisher Scientific). Nucleotide sequences were sourced from NCBI, and primer specificity was assessed using the BLAST tool (BLASTN 2.17.0+; Table 2). The ct values were determined using PikoReal 2.2 software. Gene expressions were normalized to the reference genes H3 histone family member 3A (*H3-3A*) and glyceraldehyde-3-phosphate dehydrogenase (*GAPDH*).

### 4.4. Caspase 3/7 Assay

For the Apo-One^®^ Homogeneous Caspase 3/7 Assay (Promega, Madison, WI, USA), the cultivation was performed with Ø 2 mm (KAI Medical) retinal explants (*n* = 7/group) in a 96-Well plate (Thermo Fisher Scientific Inc.). Each well contained 100 µL of medium. Samples were otherwise cultivated as described under 4.1. After four days, the explants were frozen at −80 °C for 50 min and then thawed at 37 °C for 30 min to enhance cell lysis. The assay reagent was applied 1:1 in each well and the RFU were measured on a shaker with a multiplate reader after 1.5 h (SpectraMax i3x, Molecular Devices, San Jose, CA, USA).

### 4.5. Statistical Analysis

Regarding the immunohistological examination and the caspase 3/7 assay, all groups were compared by one way ANOVA, followed by post hoc Tukey HSD test (Statistica V13, StatSoft, Hamburg, Germany). Results were shown as mean ± SEM. RT-qPCR data were evaluated using REST 2009 V2.0.13 (Qiagen, Hilden, Germany). Here, the median was shown with quartiles and deviations (minimum/maximum). All groups were compared with each other. *p*-values were determined as follows: * *p* < 0.050, ** *p* < 0.010, and *** *p* < 0.001.

## 5. Conclusions

**Figure 8:**
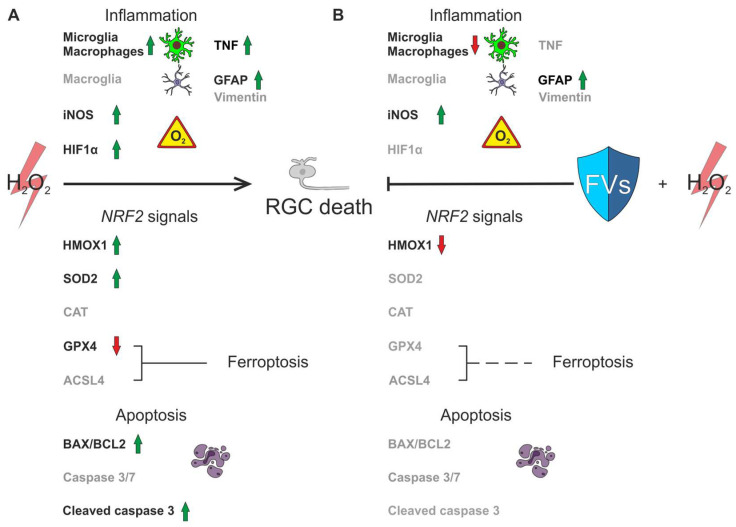
Graphical summary regarding the damage of H_2_O_2_ (**A**) as well as the protective effects of FVs (**B**). A significant loss of RGCs was observed after oxidative stress, which was no longer detectable in FVs pre-treated samples. H_2_O_2_ led to an increased number of microglia and macrophages, which was reversed by FVs. *TNF* expression was upregulated by H_2_O_2_ and the addition of FVs reversed this effect to control expression. Stress-induced upregulation was evident on transcriptional level (*GFAP*), which was mitigated only with higher concentrations of FVs. Both oxidative (*NOS2*) and hypoxic (*HIF1A*) markers were upregulated after H_2_O_2_ exposure and reversed by high FVs concentrations. H_2_O_2_ increased the *BAX*/*BCL2* ratio as well as the number of cl. casp. 3 positive cells, indicating apoptotic signaling. FVs mitigated this alteration. No effect was visible regarding the caspase 3/7 activity. The Nrf2 signaling pathways revealed an upregulation of *HMOX1* and *SOD2* through H_2_O_2_, while it inhibited *GPX4* expression, which is also related to ferroptosis. FVs treatment reversed these effects. *CAT* expressions were unchanged in all groups. Green arrow = upregulated; red arrow = downregulated; greyed out = not regulated; connection line = triggered; dashed connection line = no longer triggered.

In this study, the potential neuroprotective effect of FVs was investigated in a porcine *ex vivo* retina organ culture model. FVs could prevent RGC loss by affecting various signal cascades. It reduced inflammatory accumulation of microglia and macrophages and lowered oxidative and hypoxic stress signaling. Also, it regulated specific anti-oxidative and anti-ferroptotic genes. An apoptotic response after oxidative stress could be detected on mRNA level and within the cell count. Given the remarkable expression pattern of Nrf2-associated genes, we hypothesize a significant involvement of this regulatory signaling pathway. The analysis of ferroptotic markers revealed a notable role of FVs in its inhibition on mRNA level (Figure 8).

Overall, this study provides valuable insights into the molecular mechanisms driving glaucomatous degeneration and emphasizes the neuroprotective effect of FVs on RGCs in an *ex vivo* retina organ culture model. These findings support the potential of FVs as a novel therapeutic approach, paving the way for future clinical research and treatment strategies for glaucoma.

## Figures and Tables

**Table 1:** Primary and secondary antibodies used for immunohistology.

Primary Antibodies				Secondary Antibodies		
Antibody	Source	Company	Dilution	Antibody	Company	Dilution
Anti-ACSL4	Rabbit	Invitrogen	1:100	Donkey anti-rabbit Alexa Fluor 555	Invitrogen	1:500
Anti-cleaved caspase 3	Rabbit	Sigma Aldrich	1:300	Donkey anti-rabbit Alexa Fluor 555	Invitrogen	1:500
Anti-Iba1	Chicken	Synaptic Systems	1:500	Donkey anti-chicken Alexa Fluor 488	Jackson Immuno Research	1:500
Anti-GFAP	Chicken	Millipore	1:400	Donkey anti-chicken Cy3	Millipore	1:500
Anti-GPX4	Goat	Thermo Fisher	1:100	Donkey anti-goat Alexa Fluor 488	Jackson Immuno Research	1:500
Anti-RBPMS	Rabbit	Millipore	1:200	Donkey anti-rabbit Alexa Fluor 555	Invitrogen	1:500
Anti-vimentin	Mouse	Sigma-Aldrich	1:500	Donkey anti-mouse Alexa Fluor 488	Invitrogen	1:500

**Table 2:** Sequences of primers used for RT-qPCR. Fwd = forward; rev = reverse; acc. no. = accession number.

Gene	Primer Fwd (5′-3′) Primer Rev (5′-3′)	GenBank Acc. No.	Amplicon Size (bp)
*BAX*	AGCGCATTGGAGATGAACTG AAGTAGAAAAGCGCGACCAC	XM_003127290.5	157
*BCL2*	GACTTCTCTCGTCGCTACCG CCGAACTCAAAGAAGGCCAC	XM_021099593.1	155
*CAT*	GAGCCTACGTCCTGAGTCTC TTGATGCCCTGGTCAGTCTT	NM_214301.2	171
*GAPDH*	CCCCTTCATTGACCTCCACT CAGCATCGCCCCATTTGATT	AF017079.1	167
*GFAP*	GGAGAAGCCTTTGCTACACG TCTTCACTCTGCCTGGGTCT	NM_001244397.1	170
*GPX4*	CATGCACGAATTCTCAGCCA AGGCCAGAATCCGTAAACCA	NM_214407.1	179
*H3-3A*	ACTGGCTACAAAAGCCGCTC ACTTGCCTCCTGCAAAGCAC	NM_213930.1	232
*HIF1A*	ACTTCTGGGCCGCTCAATTT TCCACCTCTTTTGGCAAGCA	NM_001123124.1	133
*HMOX1*	GGCTGAGAATGCCGAGTTCA GTGGTACAAGGACGCCATCA	NM_001004027.1	88
*ITGAM*	AGAAGGAGACACCCAGAGCA GTAGGACAATGGGCGTCACT	XM_021086380.1	169
*NOS2*	CGCTGTCGTGGAGATCAATG GACCAACCAAATCCAGTCGG	NM_001143690.1	157
*RBPMS*	CGAGAAGGAGAACACCCCGAAC CAAAAGACAGGTGTGTTGGGC	XM_003133393.4	549
*SOD2*	CAGCTCGAGCAGGAATCTGG CCATAGTCGTACGGCAGGTC	NM_214127.2	87
*TNF*	GCCCTTCCACCAACGTTTTC CAAGGGCTCTTGATGGCAGA	NM_214022.1	97
*TUBB3*	CAGATGTTCGATGCCAAGAA GGGATCCACTCCACGAAGTA	NM_001044612.1	164

## Data Availability

The datasets generated during and/or analyzed during the current study are available from the corresponding author on reasonable request.

## Abbreviations

The following abbreviations are used in this manuscript:

ACSL4	Acyl-CoA-synthetase long-chain family member 4
AKT	Protein kinase B
AMD	Age-related macular degeneration
AP-1	Activator protein 1
ARE	Antioxidant response element
BAK	Bcl-2 homologous antagonist
BAX	Bcl-2-associated X protein
BCL2	B-cell lymphoma 2
CAT	Catalase
cDNA	Complementary desoxyribonucleic acid
Cl. casp. 3	Cleaved caspase 3
CO_2_	Cobalt chloride
d	Day
DAPI	4′,6-Diamidin-2-phenylindol
ERK	Extracellular signal-regulated kinase
FVs	*Fucus vesiculosus*
GFAP	Glial fibrillary acidic protein
GPX4	Glutathione peroxidase 4
GCL	Ganglion cell layer
h	Hour
H_2_O_2_	Hydrogen peroxide
HIF1A	Hypoxia-inducible factor 1-alpha
HIFs	Hypoxic inducible factors
HMOX1	Heme oxygenase 1
HSP27	Heat shock protein 27
Iba1	Ionized calcium-binding adapter molecule 1
IHC	Immunohistochemistry
INL	Inner nuclear layer
iNOS	Inducible nitic oxide synthase
IOP	Intraocular pressure
IPL	Inner plexiform layer
ITGAM	Integrin alpha M
MAPK	Mitogen-activated protein kinase
min	Minute
mL	Millilitre
mm	Millimetre
mRNA	Messenger ribonucleic acid
mtDNA	Mitochondrial desoxyribonucleic acid
mTOR	Mammalian target of rapamycin
NF-κB	Nuclear factor kappa-light-chain-enhancer of activated B cells
NGF	Nerve growth factor
NOS2	Nitric oxide synthase 2
NRF2	Nuclear factor erythroid 2-related factor 2
ONL	Outer nuclear layer
PGC-1α	Peroxisome proliferator-activated receptor gamma coactivator 1-alpha
PI3K	Phosphoinositid-3-kinase
RBPMS	RNA-binding protein with multiple splicing
RFU	Relative fluorescence unit
RGC	Retinal ganglion cell
ROS	Reactive oxygen species
RT-qPCR	Quantitative real-time polymerase chain reaction
SARS-CoV-2	Severe acute respiratory syndrome Coronavirus 2
SEM	Standard error of mean
SOD2	Superoxide dismutase 2
TNF	Tumor necrosis factor
TUBB3	Tubulin beta 3 class III
µg	Microgram
µL	Microlitre
µM	Micromolar
µm	Micrometre
